# Comparison of primary human gingival fibroblasts from an older and a young donor on the evaluation of cytotoxicity of denture adhesives

**DOI:** 10.1590/1678-7757-2016-0594

**Published:** 2018-02-15

**Authors:** Aline S. L. Santoro Soares, Miriam Zaccaro Scelza, Janaína Spoladore, Marcos Antônio Gallito, Felipe Oliveira, Rita de Cássia Martins Moraes, Gutemberg Gomes Alves

**Affiliations:** 1 Universidade Federal Fluminense Universidade Federal Fluminense Faculdade de Odontologia Laboratório Experimental de Cultura Celular NiteróiRio de Janeiro Brasil Universidade Federal Fluminense, Faculdade de Odontologia, Laboratório Experimental de Cultura Celular, Niterói, Rio de Janeiro, Brasil.; 2 Universidade Federal Fluminense Universidade Federal Fluminense Hospital Universitário Antônio Pedro Unidade de Pesquisa Clínica NiteróiRio de Janeiro Brasil Universidade Federal Fluminense, Hospital Universitário Antônio Pedro, Unidade de Pesquisa Clínica, Niterói, Rio de Janeiro, Brasil.; 3 Universidade Federal Fluminense Universidade Federal Fluminense Faculdade de Odontologia NiteróiRio de Janeiro Brasil Universidade Federal Fluminense, Faculdade de Odontologia, Niterói, Rio de Janeiro, Brasil.; 4 Universidade Federal Fluminenes Universidade Federal Fluminenes Instituto de Biologia Departamento de Biologia Celular e Molecular NiteróiRio de Janeiro Brasil Universidade Federal Fluminense, Instituto de Biologia, Departamento de Biologia Celular e Molecular, Niterói, Rio de Janeiro, Brasil.

**Keywords:** Older adult, Fibroblasts, Dental prostheses, Growth factors, Biocompatibility, Interleukin

## Abstract

Denture adhesives (DA) improve the retention and stability of ill-fitting dentures, especially for older adults. These materials should be biocompatible, i.e., they cannot cause undesired biological responses and be non-cytotoxic to oral tissues. However, *in vitro* testing of DA biocompatibility employing primary cell culture may possibly be affected by other factors, such as the donor age.

**Objective:**

To compare the cytotoxicity of three different denture adhesives when assessed in primary gingival fibroblasts from a young donor or from an older donor, as well as the release of the basic fibroblast growth factor (bFGF), and the inflammatory response marker interleukin-6 (IL-6).

**Material and Methods:**

Gingival fibroblasts isolated from a 30- and a 62-year-old donor were assayed for proliferation (1-7 days) and sensitivity to latex (positive control). Fibroblasts were indirectly exposed to Corega Ultra (cream), Corega powder and Fixodent Original for a 24 h period and assayed by XTT and Crystal Violet tests. The release of IL-6 and bFGF by exposed cells was determined by ELISA.

**Results:**

While cells from the young donor presented higher cell growth after 7 days, the sensitivity to increasing concentrations of latex extracts was very similar between young and older cells. Both XTT and CVDE detected no difference between the DA and the control group. All materials induced higher levels of IL-6 and bFGF compared to control. Cells from the older donor exposed to Corega Ultra released lower levels of cytokine and growth factor.

**Conclusions:**

All materials were considered non-cytotoxic, but affected cytokine and growth factor release. The biological differences found between fibroblasts from both donors could be due to individual or age-related factors. The authors suggest the use of cells from older donors on studies of dental products aimed at older patients, to better simulate their physiological response.

## Introduction

Dentists often recommend the use of denture adhesives (DA) to correct prosthetic failures and to improve the comfort and function of dentures worn by patients presenting ridge resorption, decreased salivary flow, poor neuromuscular coordination, a low denture adaptability, and cognitive impairment^17^. The use of DA increases masticatory function by enabling the retention and stability of dentures, minimizing the likelihood of mechanical dislodgement of dentures and consequently decreasing mucosal irritation and ulceration^13^. However, literature reports the possibility of lesions in the oral mucosa due to misuse of DA^1^^,^^16^, where the very composition of materials may cause adverse responses, such as tissue irritation. These are among the main reasons for pre-clinical and clinical safety assessments of such materials. Literature presents varied reports of *in vitro* cytoxicity evaluations in commonly used denture adhesives during pre-clinical assessments^5^^,^^18^. Gomes, et al.^11^ (2011) demonstrated that 10 different DAs induced similar dose- and time-dependent cytotoxic profiles when exposed to a murine fibroblast cell line (L929), affecting cell survival and morphology at different levels as assessed by direct and indirect assays – including Corega cream and powder (Stafford- Miller, Dungarvan, Waterford, Ireland). Another study using the same cell line confirmed that different brands of denture adhesives may present *in vitro* toxicity^1^, possibly increasing the risk of mucosal inflammation in denture wearers.

However, several factors can influence the reliability of results or limit the extrapolation to clinical settings during *in vitro* cell-based tests. Studies indicate that tests involving primary cell culture may provide more useful data to clinicians when compared to immortalized cell lines (often from tumor origin), since primary cells present the natural ploidy, the same regulation of gene expression, the response to stress and other biological parameters observed in humans, *in vivo*^7^^,^^8^. In this regard, other studies have already employed primary human cell models, such as human gingival fibroblasts, to assess the cytotoxicity of several dental materials^12^, including the widely used DA Fixodent Fresh^10^. Thus, we demonstrated that primary human oral mucosal cells may provide more valuable information in toxicity screening of denture adhesives when compared to animal-related cell lines^5^.

Human gingival fibroblasts are among the most abundant resident cells from the oral mucosa, they feature a higher ability for scarless wound healing when compared to skin fibroblasts^20^, mostly due to a differential release of growth factors, such as TGF-Beta, connective tissue growth factor (CTGF) and basic fibroblast growth factor (bFGF). Besides producing the extracellular matrix (ECM) in oral connective tissues, human gingival fibroblasts express the cell surface proteins CD14, TLR4, and MD-2, and produce pro-inflammatory cytokines such as interleukin 6 (IL-6)^3^, indicating an important immunomodulatory role in response to stress and diseases such as periodontitis. Therefore, these cells may represent interesting models for *in vitro* assessment of dental materials by also allowing the investigation of the release of growth factors and cytokines that affect biocompatibility. A cytokine that has long been suggested as a parameter for the evaluation of the biological activity under nontoxic experimental conditions of dental materials *in vitro*^26^ is IL-6, being a representative of stress and immune response. Similarly, other *in vitro* dental materials biocompatibility studies have also investigated the release of bFGF^6^, mostly due to its effects on proliferation, differentiation and extra-cellular matrix production of cells involved in wound healing and response to stress^4^.

However, when testing materials designed for specific groups such as geriatric patients (e.g. denture fixatives/adhesives), other factors regarding the cell test system could impose limitations for replicating the clinical conditions. It has been demonstrated that cells from older adults may show genomic instability, telomere attrition, epigenetic alterations, loss of proteostasis, deregulated nutrient-sensing, mitochondrial dysfunction, stem cell exhaustion and altered intercellular communication^15^^,^^24^. For instance, human gingival fibroblasts from older adults presents altered protein synthesis and cell cycle, as well as a reported lower sensitivity to chemical stress^2^ pulp cell HPC, periodontal ligament fibroblast HPLF^5^. However, whether cells from older adults respond differently to materials destined for geriatric care such as dental adhesives remains unanswered, since there are reports of senescent cells exhibiting functional alterations that could affect metabolic activity and the response to age-related diseases^24^. Therefore, it is possible that cells from young and older donors perform differently during *in vitro* evaluations of biocompatibility.

Thus, the objective of this study were: 1) to compare the standardized cytotoxicity evaluation^14^ of three different denture adhesives with wide applicability and with previously reported cytotoxicity tests [Fixodent Original (Procter & Gamble, Cincinnati, Ohio, USA), Corega Ultra Cream (Stafford- Miller, Dungarvan, Waterford, Ireland) and Corega Powder (Stafford- Miller, Dungarvan, Waterford, Ireland)], when assessed in gingival fibroblast primary cells from either a young and an older donor, and 2) to investigate the impact of the exposure to the materials on the release of an important fibroblast growth factor, basic FGF, and on the inflammatory response marker IL-6, in those cells.

## Material and methods

### Collection of human gingival fibroblasts

This work was part of a project approved by the Antonio Pedro Hospital Research Ethics Committee, protocol no. CAAE 50316715.2.0000.5243. Informed consent was obtained from all participants included in the study.

Primary cells were collected from a 62-year old woman and from a 30-year old woman. Both participants were patients of the Periodontics Clinic at the Fluminense Federal University, who met the following criteria: subjects indicated for surgery that allowed the collection of a gingival fragment without affecting the original surgical plan, had no chronic disease, made no continuous use of drugs and had no gingival bleeding.

After collecting the gingival tissue, the isolation of fibroblasts was performed according to a previously described protocol^2^. Fragments were immersed on polypropylene tubes containing the culture medium (Dulbecco's Modified Eagle's Medium – DMEM) with 3% antibiotics and washed with Phosphate Buffered Saline (PBS) in a sterile hood. After washing, the fragments were immersed in 70% ethylic alcohol for 1 min and washed again in PBS. To separate connective from epithelial tissue, the fragments were sectioned with a scalpel into sections of approximately 2 mm. The connective tissue fragments were treated with trypsin for enzyme digestion, with final inactivation by the addition of Fetal Bovine Serum (FBS). The digested sections were transferred to 6-well cell culture plates and allowed to adhere for 3 minutes. After this period, 1 mL of 1% antibiotic was added to each well and incubated at 37°C for 48 hours. Finally, the fragments were removed from the plate and the cell culture was established according to standard protocols for adhered cells. The resulting cells presented fibroblast morphology (elongated fusiform nuclei organized in a parallel pattern), and positive staining for vimentin and type I collagen, markers of fibroblast origin cells^9^.

### Cell culture

Cells at the second passage were cultivated at 37°C under 5% CO_2_ in DMEM containing 10% fetal bovine serum (GIBCO/Invitrogen, Grand Island, Nebraska, USA) and 1% antibiotic and antimitotic. The cultures were then seeded at a density of 3×10^4^ cells mL^-1^ in wells of a 96-well plate, followed by incubation for 24 h at 37°C, under 5% CO_2_. The cells were exposed to the test samples by replacing 200 μL of the medium in each well with 200 μL of one of the extracts described previously, followed by incubation for 24 h.

### Cell growth assay

A cell growth curve was performed for each of the cell systems (gingival primary fibroblasts from young and older adult). For each group, 10^4^ cells/well were seeded and incubated at 37°C/5% CO_2_ on DMEM added with 10% fetal bovine serum, for 1, 2, 4 or 7 days. Culture media was changed in alternate days. The density of cells was assessed by Crystal Violet Dye Exclusion assay (CVDE), correlating the number of cells with the intensity of dye adhered to DNA, as measured by the optical density at 540 nm (O.D._540_) on a Synergy II Microplate Reader (Biotek Inst., Winooski, Vermont, USA).

### Cell sensitivity assessment

A dose-response assay was performed with both cell systems to assess the cell sensitivity to cytotoxic substances, they were exposed to increasing concentrations of latex extract, a well-known positive control for cytotoxicity of dental materials^19^. Briefly, 10^4^ cells from each group were seeded in quintuplicates into 96-well culture plates and incubated for 24 h at 37°C/5% CO_2_ on DMEM added with 10% fetal bovine serum. Subsequently, the culture medium was removed and replaced by extracts produced by 24-hour incubation of increasing proportions of latex fragments in DMEM (0, 2, 10, 20, 50, 100, 150 and 200 mg/mL) at 37°C. Cell viability assessment was performed by XTT assay (In Cytotox kit, Xenometrix, Nordrhein-Westfalen, Germany).

### Cytotoxicity evaluation of denture adhesives

#### Sample preparation

**Figure 1:**
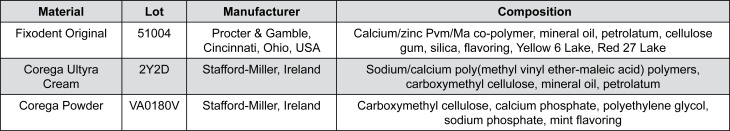
Description of the denture adhesives

Samples of the three denture adhesives Fixodent Original (Procter & Gamble, Cincinnati, Ohio, USA), Corega Ultra Cream (Stafford- Miller, Dungarvan, Waterford, Ireland) and Corega Powder (Stafford-Miller, Dungarvan, Waterford, Ireland) - Figure 1 - were prepared according to international standards for material testing^14^. Briefly, 0.2 g of each material was immersed in 1 mL of DMEM and incubated for 24 h at 37°C. Due to the high viscosity of the materials, three centrifugation steps were performed (10 min at 5500 rpm) until a clear and non-solid extract was obtained. Extracts were similarly produced with latex fragments (positive control), and high-density polystyrene beads (negative control).

#### Cytotoxicity assay

Cells were seeded into 96-well plates at a density of 10^4^ cells/well, and subcultured by 24 h at 37°C/5% CO_2_, until subconfluence. The cell culture medium was removed from each well and substituted by one of the previously described extracts [Fixodent Original (Procter & Gamble, Cincinnati, Ohio, USA), Corega Ultra Cream (Stafford- Miller, Dungarvan, Waterford, Ireland) and Corega Powder (Stafford- Miller, Dungarvan, Waterford, Ireland)], positive control or negative control, or fresh DMEM (blank group), and incubated for 24 hours.

The cell viability of the gingival fibroblasts from the young and older donors exposed to the different extracts was assayed by a test involving sequential analysis (i.e., using the same culture) for metabolic activity (XTT test) and cell density (CVDE test). The assays were performed using a commercial kit (In Cytotox - Xenometrix, Nordrhein-Westfalen Germany), allowing an accurate correlation of the results.

The XTT test evaluates the capacity of the enzyme succinate dehydrogenase to convert the water-soluble XTT tetrazolium salt to soluble formazan crystals, which present an orange color, directly correlated to the metabolic activity of viable cells. The optical density (OD) of each well was measured at 480 nm using a Synergy II microplate reader (Biotek Inst, Winooski, Vermont, USA), and the metabolic activity of viable cells after exposure to each material was reported in relation to the blank group (unexposed cells), which was assumed to show 100% viability.

The CVDE assay was performed in the same cells, after they were washed with Phosphate Buffered Saline (PBS) using the Crystal Violet dye, which stains the DNA and provides a measure of cell density. After washing and removing the excess dye, the OD at 540 nm was directly proportional to the number of adherent cells in each well and expressed as a percentage of the blank group.

### Measurement of IL-6 and bFGF

Gingival fibroblasts from both donors were seeded into 96-well plates at a density of 10^4^ cells/well, subcultured for 24 h at 37°C/5% CO_2_ until subconfluence, and subsequently exposed to extracts of each tested material for 24 hours. Tissue Culture Polystyrene (TCPS) was used as a negative control to determine basal release of the cytokine and growth factor. The experiment was performed in quintuplicates. The culture supernatants were collected after 24 hours of incubation and pooled. Fibroblast Growth Factor and IL-6 were measured by Enzyme-Linked Immunosorbent Assay (ELISA), using commercially available Human FGF-basic and Human IL-6 ELISA Development Kits (PeproTech, Rocky Hill, New Jersey, USA), with sensitivity range of 63-4000 pg/mL for FGF and 24-1500 pg/mL for IL-6. The procedure followed the conditions and recommendations of the manufacturer. The plates were coated with capture antibody overnight at room temperature. Then, they were washed and blocked with 1% Bovine Serum Albumin (BSA) on PBS for an hour. The samples and standards were added and incubated for two hours. Detection antibodies were added for immunological sandwich formation, finally, peroxidase and ABTS Liquid Substrate were added to develop color. The OD value was measured in a Synergy II microplate reader (Biotek Inst, USA) on kinetic mode (11 readings) for 20 minutes at 405 nm. The reaction was stopped with a 5% sodium dodecyl sulfate (SDS) solution, and the final reading obtained at 405 nm with wavelength correction set at 650 nm.

Results were subsequently normalized by the estimated density of cells in each well, as measured by the CVDE method, through the reading of OD at 540 nm after DNA staining with Crystal Violet Dye.

### Statistical analysis

For the ELISA assays, sample concentrations were established by comparison with standard curves generated from standards provided by kits. ELISA and cytotoxicity assay results were evaluated for normal distribution by D'Agostino's K-squared test, and by Kruskal-Wallis nonparametric test with Dunn's post-test were employed for comparisons. For the estimation of IC_50_ (concentration capable of inhibiting 50% of cells), results were plotted on a logarithmical scale and a logistic regression was performed using the GraphPad Prism 6.0 software (GraphPad Inc., USA). The growth curves of cells from both donors were compared through Student's t-test. For all statistical analysis an alpha error of 5% was considered, therefore, a p<0.05 was considered significant.

## Results

**Figure 2:**
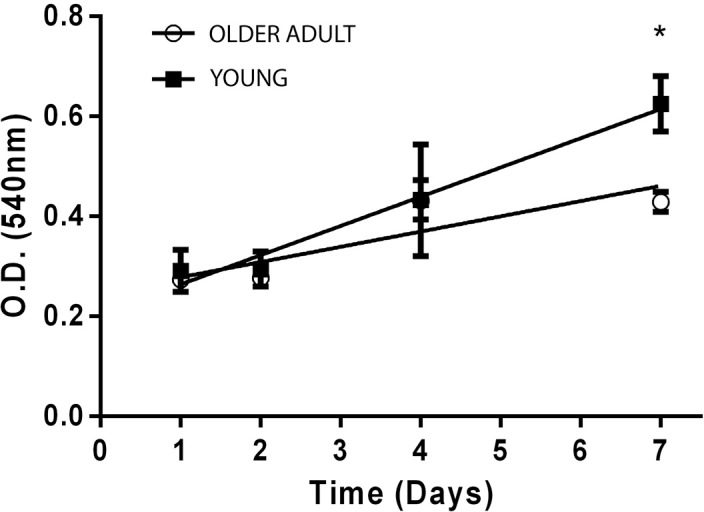
Growth curves for gingival fibroblasts from the young (black squares) and the older (white circles) donor, based on cell density as measured by the optical density (O.D.) of DNA staining by crystal violet dye. *Statistically significant difference between groups (P<0.05)

Figure 2 shows the assessment of cell growth during the first week of culture for fibroblasts from the both donors. While no difference was found between both cultures within 24 hours, a significant increase of 46% in proliferation was observed for fibroblasts of the young donor when compared to cells from the older donor (t-test, p<0.05) by the 7^th^ day after cell seeding.

Fibroblasts from both donors exposed to increasing concentrations of latex extract, as the positive cytotoxicity control, behaved similarly, with IC_50_ values of 8.95 and 9.16 mg/mL, respectively.

**Figure 3:**
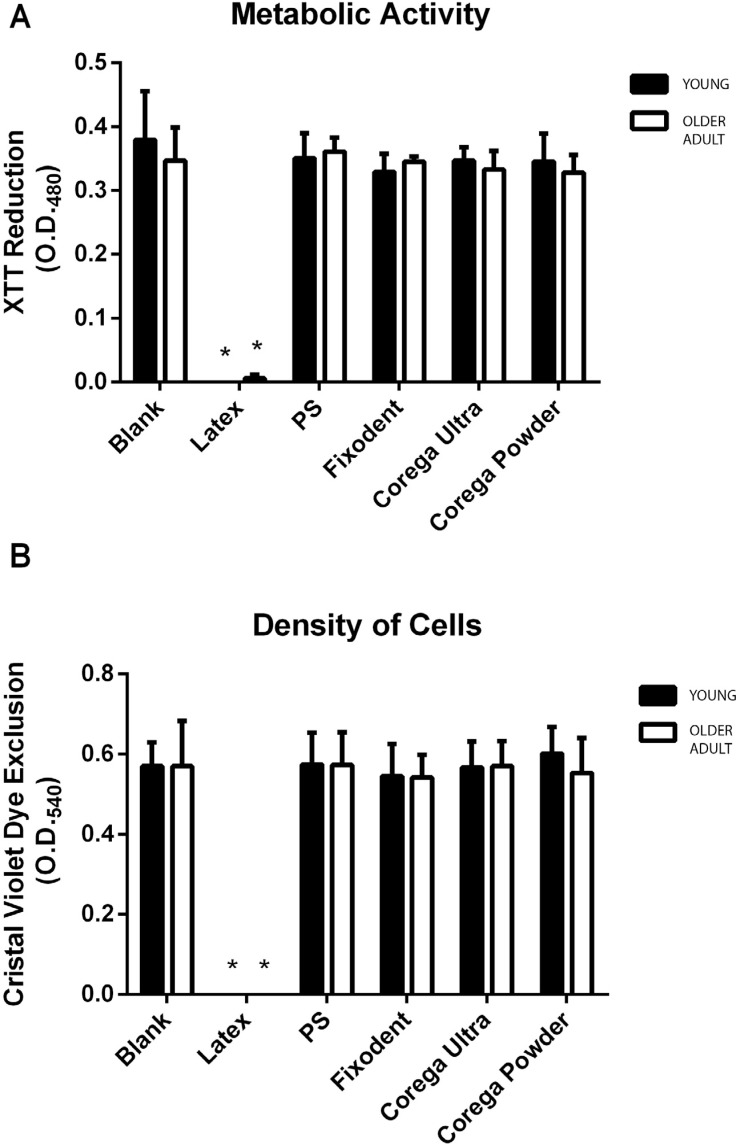
Metabolic activity and cell density were measured by XTT assay (A) and CVDE test (B) performed with gingival fibroblasts from the young (black) and older (white) donors, against the denture adhesives Fixodent, Corega Ultra or Corega powder. Bars indicate mean±SD of two independent assays in quintuplicates, reported as the optical densities measured at 480 (O.D.480) or 540 nm (O.D.540). Cells exposed to culture medium were used as blank, polystyrene beads (PS) as a negative control and latex extracts as a positive control. *Significantly different from all other groups (P<0.05)

Figure 3 shows the cytotoxicity results of the three denture adhesives. As expected, the positive control caused cell death of almost 100% (Figures 3A and 3B), and the negative control presented no significant difference from the blank group (unexposed cells). None of the tested denture adhesives presented cytotoxicity to human gingival fibroblasts after 24 h exposure in both cell viability parameters tested (metabolic activity and cell density), regardless of the cell origin (young or older donors). No statistically significant difference was observed between the tested materials, or between the materials and the control group (non-parametric analysis of variance, p>0.05).

**Figure 4:**
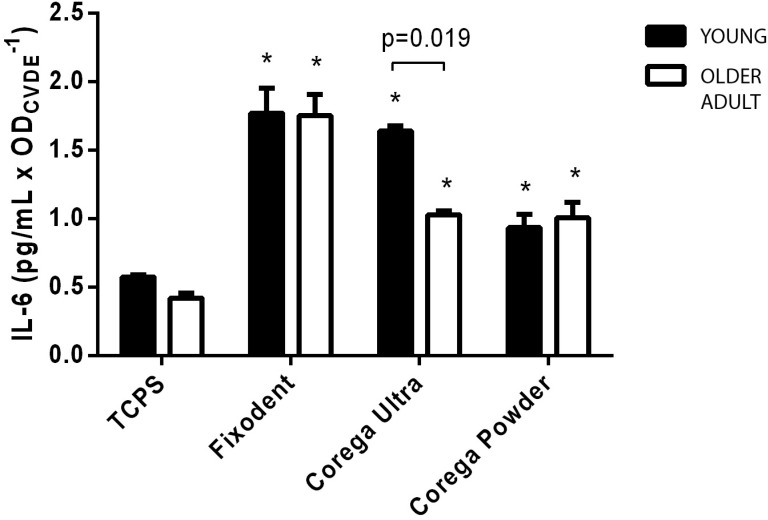
Release of IL-6 in the culture medium by gingival fibroblasts from the young (black) or the older (white) donor in contact with Fixodent, Corega Ultra or Corega powder. Data were normalized against the density of cells, as measured by the optical density at 540 nm during a CVDE assay (ODCVDE). Tissue culture polystyrene (TCPS) was considered the negative control. The SD values are indicated by error bars. *Significantly different from the control group (Blank) at each age-dependent cell type (P<0.05). Significance bars indicate differences between age-dependent cell types (unpaired t-test)

Figure 4 shows the results for IL-6 release levels in culture medium after 24 hours of exposure to extracts of the three denture adhesives. While the control group (exposed to tissue culture polystyrene, TCPS) induced low levels of IL-6 release, exposure to Fixodent led to a fourfold increase on the mean level of this cytokine in fibroblasts from both donors (p<0.05), and Corega Powder induced a twofold increase on IL-6. Exposure to Corega Ultra extracts also caused a significant increase (P<0.05) on young donor fibroblasts, while this increase was 38% lower for cells from the older donor.

**Figure 5:**
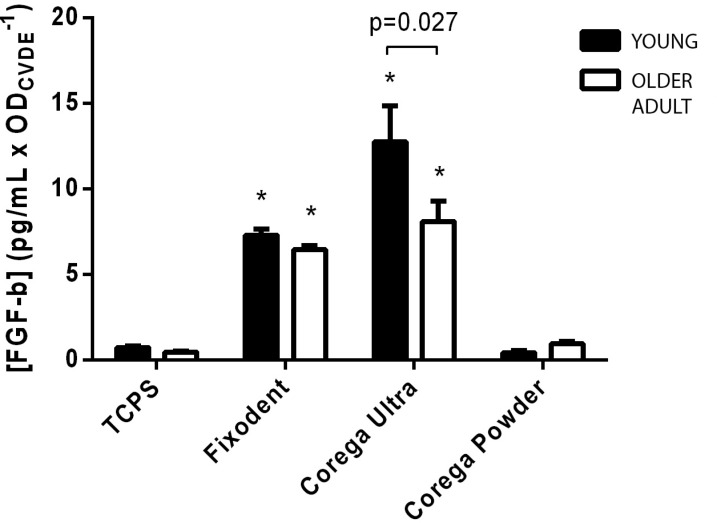
Release of bFGF in the culture medium by gingival fibroblasts from the young (black) or the older (white) donor in contact with Fixodent, Corega Ultra or Corega powder. Data were normalized against the density of cells, as measured by the optical density at 540 nm during a CVDE assay (ODCVDE). Tissue culture polystyrene (TCPS) was considered the negative control. The SD values are indicated by error bars. *Significantly different from the control group (Blank) at each age-dependent cell type (P<0.05). Significance bars indicate differences between age-dependent cell types (unpaired t-test)

Figure 5 shows a similar assessment of the mean levels of bFGF after exposure to TCPS (Control) or to extracts of each tested material. Once again, a small release of this growth factor in fibroblasts of the control group was observed, which was significantly increased by exposure to Fixodent and Corega Ultra (p<0.05). However, the increase in bFGF induced by Corega Ultra was also observed in cells from the older donor, which was significantly different from the control and young donor groups (P<0.05). Exposure to extracts of Corega Powder, as observed with IL-6, did not induce significant differences in the release of bFGF when compared to the Control group (p>0.05).

## Discussion

**Figure 6:**
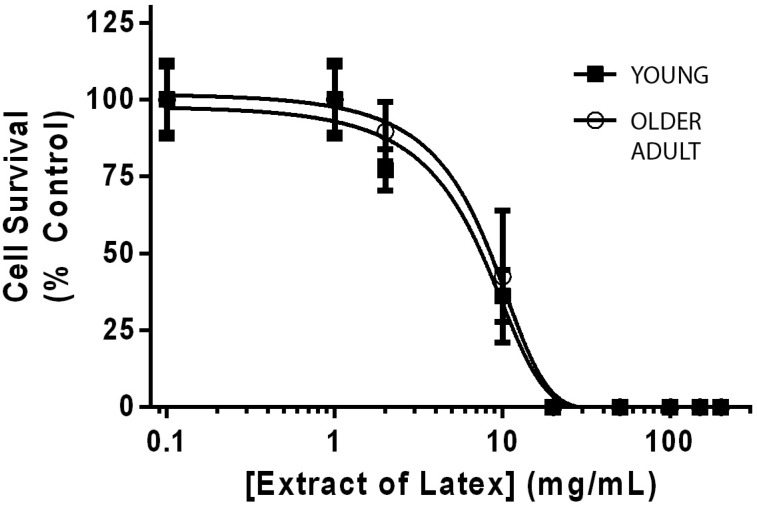
Dose-response of gingival fibroblasts from the young (black squares) and the older (white circles) donor to increasing doses of latex extracts (mg/mL), as measured by XTT assay. Results are reported as mean±SD of quintuplicates, normalized as a percentage of the control group (unexposed cells). Latex concentration is represented on a log 10 scale. The lines represent the logistic regression fit for each curve, performed with Graphpad Prism 6.0

Evidence suggests that fibroblasts from older individuals are likely to be more sensitive to adverse effects, since age-related frailty can influence cellular senescence, loss of telomeric structures, mitochondrial activity, production of reactive oxygen species and DNA repair capability^29^. However, in this study we showed that the sensitivity to a well-known cytotoxic system (latex extracts) is not affected by the age of the primary gingival fibroblasts donor, having very similar inhibitory curves (Figure 6), and IC_50_ values similar to those described for other primary cell models^19^.

Similarly, the age of the donor did not affect the outcome of cytotoxicity tests of the denture adhesives Fixodent, Corega Powder and Corega Ultra, which had already been assessed by other authors^10^^,^^11^, using different cell models. Our results for the combined XTT/CVDE tests showed that all tested DAs are non-cytotoxic regardless of the age of the donor (Figure 3). According to ISO 10933-5:2009^14^, materials are considered non-cytotoxic when 70% or more of viable cells is reached, therefore, all the three materials tested are safe for general consumer use regarding cytotoxicity. We note that the cytotoxicity assay was performed with a 24-hour exposure time, as recommended by ISO 10993-5 and followed by several cytotoxicity studies. Figure 2 shows that the proliferation of cells from both donors was very similar up to the 4^th^ day of culture. However, since proliferation was significantly different by the 7^th^ day of culture we did not discard the possibility that the age of the donor could affect the results of assays if longer exposures/culture times were used.

However, different responses were found when assessing the release of important markers of fibroblast behavior and inflammatory reaction, depending on both the tested material and the age of the donor. Regarding IL-6, a pro-inflammatory cytokine involved in several processes including response to trauma and infection^21^, we found that all three DAs induced significantly higher levels (p<0.05) when compared to control, Fixodent presented the highest level. Trubiani, et al.^28^ (2012) reported an increased release of IL-6 by human gingival fibroblasts when exposed to denture base acrylic resins, and suggested that this cytokine is a good marker for *in vitro* biocompatibility testing of dental materials. While the presence of cytokines in damaged tissue is important for the immune defense in response to pathogens, increased concentrations of IL-6 are known to delay tissue healing and contribute to bone resorption^27^, and are also associated with increased palatal inflammation in denture stomatitis (DS)^23^. Therefore, the present results reinforce that patients should seek the advice of the dental clinician prior to the use of adhesives, to consider the risks of prolonged exposure in subjects with DS or excessive bone resorption^1^. Additionally, evidence from literature indicates a link between IL-6 and late-life diseases and frailty, affecting the health of older adults^5^. Curiously, a significant reduction was observed in IL-6 levels induced by Corega Ultra in cells from the older donor, indicating that the impact of cytokine release on older adults' users of this DA may be lower than the predicted by the usual cell models (often of cell lines or young donor origin).

Similarly, differences were observed in the release of bFGF, an important growth factor, reported as relevant in wound healing and tissue repair by accelerating the proliferation of fibroblasts^30^. The results indicate a significant increase in bFGF release induced by both Fixodent and Corega Ultra, but no clear effect from exposure to Corega Powder was found. While this important growth factor is often related to repair and tissue regeneration by influencing the proliferation of fibroblasts and epithelial cells, the increased release of fibroblasts from otherwise healthy tissues exposed to denture adhesives must be carefully regarded, since this may also imply in a cell response induced by injury or irritation^11^, including those caused by indirect or direct contact with a material.

The model used was unable to detect differences in bFGF release between unexposed cells from young or older donors. However, previous reports indicate that the process of aging decreases the physiological concentration of bFGF in saliva^30^, which could be related to a reduction of mucosal cell proliferation and collagen secretion. This reduction could also affect structural and mechanical properties of the connective tissue extracellular matrix (ECM), which are implicated on age-related tissue stiffness^22^ changes occur in the collagen network that contribute to various pathological phenotypes in the skeletal, vascular, and pulmonary systems. The aim of this study was to investigate the consequences of age-related modifications on the mechanical stability and *in vitro* proteolytic degradation of type I collagen. Analyzing mouse tail and bovine bone collagen, we found that collagen at both fibril and fiber levels varies in rigidity and Young's modulus due to different physiological changes, which correlate with changes in cathepsin K (CatK. On the other hand, during inflammatory response, the release of bFGF increases on similar levels for both young and older individuals^30^, suggesting a role in the oral response to injuries and irritation. Therefore, a dual interpretation is raised for the possible benefits or drawbacks of the reduction of bFGF release in older cells exposed to Corega Ultra, as it may imply both a decrease of wound-healing capacity and maintenance of ECM integrity, or a lower response to irritation caused by this material. Therefore, further *in vivo* or clinical assessments of bFGF release and impact are needed to confirm its role on the safe use of denture adhesives.

One of the main limitations of this preliminary study is that the comparison was performed with a single donor for each age group. Therefore, the possibility that individual, rather than age-related factors, might have affected the outcomes of the cytotoxicity tests is not discarded. Nevertheless, the overall results indicate similarities and relevant differences in the performance of human gingival fibroblasts of a donor with advanced age when compared to a younger donor, during the *in vitro* biological evaluation of denture adhesives. While simple endpoints such as cytotoxicity were unaffected, this result could be altered with longer experimental times, since the proliferation of fibroblasts from older donors was affected after seven days (Figure 2). Furthermore, the results indicate that the outcomes of advanced biocompatibility assays, including protein expression, -omics studies (e.g.: genomics, proteomics or metabolomics) or the evaluation of cell-cell interactions, might be affected by the choice of cell system, which must be assessed in future studies, employing larger samples of each experimental age group. This is especially relevant if we consider the need to prioritize more relevant and mechanistic insights of the toxic pathways, instead of evaluating single isolated cytotoxicity endpoints. Thus, even though the clinical relevance of *in vitro* tests should be considered carefully, considering the results we confirm the proposed hypothesis that cells from a young and an older donor may behave differently in the evaluation of denture adhesives biocompatibility. In this context, using primary human fibroblasts from older donors for the *in vitro* testing of materials in which age-dependent factors are relevant, such as denture adhesives, could provide more clinically relevant results.

## Conclusions

While all materials were considered non-cytotoxic regardless of the age of the donor, Fixodent and Corega Ultra induced different levels of cytokine and growth factor release in fibroblasts from young and older donors. These differences suggest the relevance of using cells from older donors on studies of dental products aimed at older patients.
